# Multi-tensor diffusion abnormalities of gray matter in an animal model of cortical dysplasia

**DOI:** 10.3389/fneur.2023.1124282

**Published:** 2023-05-05

**Authors:** Paulina J. Villaseñor, David Cortés-Servín, Aylín Pérez-Moriel, Ana Aquiles, Hiram Luna-Munguía, Alonso Ramirez-Manzanares, Ricardo Coronado-Leija, Jorge Larriva-Sahd, Luis Concha

**Affiliations:** ^1^Instituto de Neurobiología, Universidad Nacional Autónoma de México Campus Juriquilla, Querétaro, Mexico; ^2^Centro de Investigación en Matemáticas, A.C., Guanajuato, Mexico; ^3^Bernard and Irene Schwartz Center for Biomedical Imaging, Department of Radiology, New York University School of Medicine, New York, NY, United States

**Keywords:** diffusion-weighted imaging, focal cortical dysplasia, gray matter, diffusion tensor imaging, immunofluorescence, epilepsy

## Abstract

Focal cortical dysplasias are a type of malformations of cortical development that are a common cause of drug-resistant focal epilepsy. Surgical treatment is a viable option for some of these patients, with their outcome being highly related to complete surgical resection of lesions visible in magnetic resonance imaging (MRI). However, subtle lesions often go undetected on conventional imaging. Several methods to analyze MRI have been proposed, with the common goal of rendering subtle cortical lesions visible. However, most image-processing methods are targeted to detect the macroscopic characteristics of cortical dysplasias, which do not always correspond to the microstructural disarrangement of these cortical malformations. Quantitative analysis of diffusion-weighted MRI (dMRI) enables the inference of tissue characteristics, and novel methods provide valuable microstructural features of complex tissue, including gray matter. We investigated the ability of advanced dMRI descriptors to detect diffusion abnormalities in an animal model of cortical dysplasia. For this purpose, we induced cortical dysplasia in 18 animals that were scanned at 30 postnatal days (along with 19 control animals). We obtained multi-shell dMRI, to which we fitted single and multi-tensor representations. Quantitative dMRI parameters derived from these methods were queried using a curvilinear coordinate system to sample the cortical mantle, providing inter-subject anatomical correspondence. We found region- and layer-specific diffusion abnormalities in experimental animals. Moreover, we were able to distinguish diffusion abnormalities related to altered intra-cortical tangential fibers from those associated with radial cortical fibers. Histological examinations revealed myelo-architectural abnormalities that explain the alterations observed through dMRI. The methods for dMRI acquisition and analysis used here are available in clinical settings and our work shows their clinical relevance to detect subtle cortical dysplasias through analysis of their microstructural properties.

## Introduction

Focal cortical dysplasias (FCDs) were first described more than five decades ago (1). They represent malformations of cortical development and are the most common anatomical lesion identified in children (and second in adults) with drug-resistant focal onset epilepsy (2–4). Disruption of cortical layering and the presence of abnormal cells (i.e., dysmorphic neurons and balloon cells) are two of their most common histological features (3). Various etiologies have been proposed for FCDs, comprising mainly genetic and environmental factors (5). However, the precise physiopathological mechanisms by which FCDs cause neuronal hyperexcitability and seizures remain a topic of active research (6–10).

Magnetic Resonance Imaging (MRI), currently the main non-invasive technique for clinically diagnosing FCDs, often shows blurring between gray and white matter junctions, cortical thinning, and cortical hyperintensity on T2-weighted and FLAIR images (11). However, between 16 and 43% of individuals with FCD present subtle and heterogeneous lesions on their scans that are notoriously difficult to detect and therefore often overlooked on conventional imaging (12, 13). Several image processing methods have been used aiming to increase the diagnostic yield of anatomical MRI. Notably, most exploit the aforementioned imaging features of FCD and provide novel contrasts or quantitative maps that increase the conspicuity of the lesions (14, 15). Recently, multi-center surface-based analyses of high-resolution anatomical images have shown promise for the detection of FCD through machine learning algorithms (13, 16, 17). Although these findings seem promising for clinical management, the development of better detection techniques are still required.

Diffusion-weighted magnetic resonance imaging (dMRI) provides a non-invasive insight to identify microstructural tissue characteristics. Although dMRI has been mostly used for the study of white matter, it has also been proven to be sensitive to features of tissue organization in gray matter (18–20). The application of dMRI for the study of cortical structure has been difficult, as its architecture is considerably more complicated than that of white matter. Notably, cortical layering differs between regions, with distinct characteristics of neuronal shapes, sizes, and their organization that form the basis of cytoarchitectonic maps, such as that of Brodmann (21, 22). Similarly, different brain areas display particular patterns of intracortical fibers that result in myeloarchitectonic maps (23–25). Within the cortex, there are radial fibers that enter and exit the cortex and tangential fibers that enable the communication between adjacent columns, and such configuration results in the presence of crossing fiber populations within imaging voxels. Representing the dMRI signal with a single tensor, as done by diffusion tensor imaging (DTI) (26) is therefore insufficient to adequately describe complex diffusion profiles in the cortex (27). On the other hand, the multi-tensor representation of the signal is a logical extension of DTI that treats the diffusion signal as a mixture of non-exchanging Gaussian diffusion processes. An implementation of multi-tensor fitting is the multi-resolution discrete-search (MRDS) (28) which provides a robust estimation of the number of fiber populations within each voxel. Fitting an arbitrary number of different orientational compartments with Gaussian profiles has been tackled by several different approaches (29–31). However, those methods enforce the same diffusivity profile for all orientational compartments. In contrast, MRDS fits a diffusion tensor for each fiber bundle, each tensor with its own individual shape (eigenvalues), compartment size, and orientation (eigenvectors), thus providing independent orientational diffusion characteristics for each bundle within each voxel. Notably, the multi-tensor representation provides sensitivity to tissue alterations and its biological interpretation is intuitive, albeit not being specific to intra- and extracellular compartments properties (32).

Here we aimed to investigate the ability of MRDS to non-invasively identify diffusion abnormalities *in vivo*, in a rodent model of cortical dysplasia. We show that the multi-tensor approach captures layer- and region-specific diffusion alterations in the cortex of experimental animals. Histological analyses provided evidence of altered myelo- architectural features.

## Materials and methods

### Animal model

All procedures were performed according to protocols approved by our institute’s ethics review board (file 111-A) and were carried out according to federal regulatory laws for animal experimentation (NOM-602-ZOO-1999). We used an animal model that induces histological abnormalities similar to those observed in FCD Type IIa (2). This is accomplished by injection of an alkylating agent to pregnant rats at the time of cortical development of the offspring (33). Eight pregnant Sprague–Dawley rats were intraperitoneally injected with either 1,3-Bis (2-chloroethyl) -1-nitrosourea (BCNU, also known as carmustine; 20 mg/kg) (n = 4) or saline solution for control (n = 4) on embryonic day 15 (E15). Resulting pups (the object of this study) were housed with their mothers until weaning in a room with a 12 h light/dark cycle with *ad libitum* access to food and water. BCNU-treated animals showed no obvious phenotypic or behavioral characteristics from their birth until their age at dMRI scanning.

### *In vivo* diffusion imaging

Rats were scanned at postnatal day 30 (BCNU: *n* = 18, 8 female; Control: n = 19, 9 female; weight range: 65 to 80 g). Imaging was performed at the National Laboratory for MRI using a 7 T Bruker animal scanner equipped with gradients with a maximum amplitude of 760 mT/m and a 2 × 2 rat head array coil. Animals were anesthetized using isoflurane (4% for induction, 1.7% for maintenance). To maintain body temperature, they were kept warm by recirculating warm water underneath the imaging bed, and their vital signs were continuously monitored. Diffusion-weighted images were acquired with diffusion sensitization in 90 different directions, each with *b* values of 670, 1,270, and 2010 s/mm^2^. (diffusion gradient pulse duration δ = 3 ms and separation Δ = 9 ms). Fifteen b = 0 s/mm^2^ images were also acquired. All acquisitions were based on a coronal two-dimensional single echo echo-planar (EPI) sequence with the following parameters: TR 2000 ms, TE 22.86 ms and spatial resolution of 0.175 × 0.175 × 1.0 mm (0.25 inter-slice gap; 24 slices). Total scan time of 19 min.

### Analysis of dMRI

**Figure 1:**
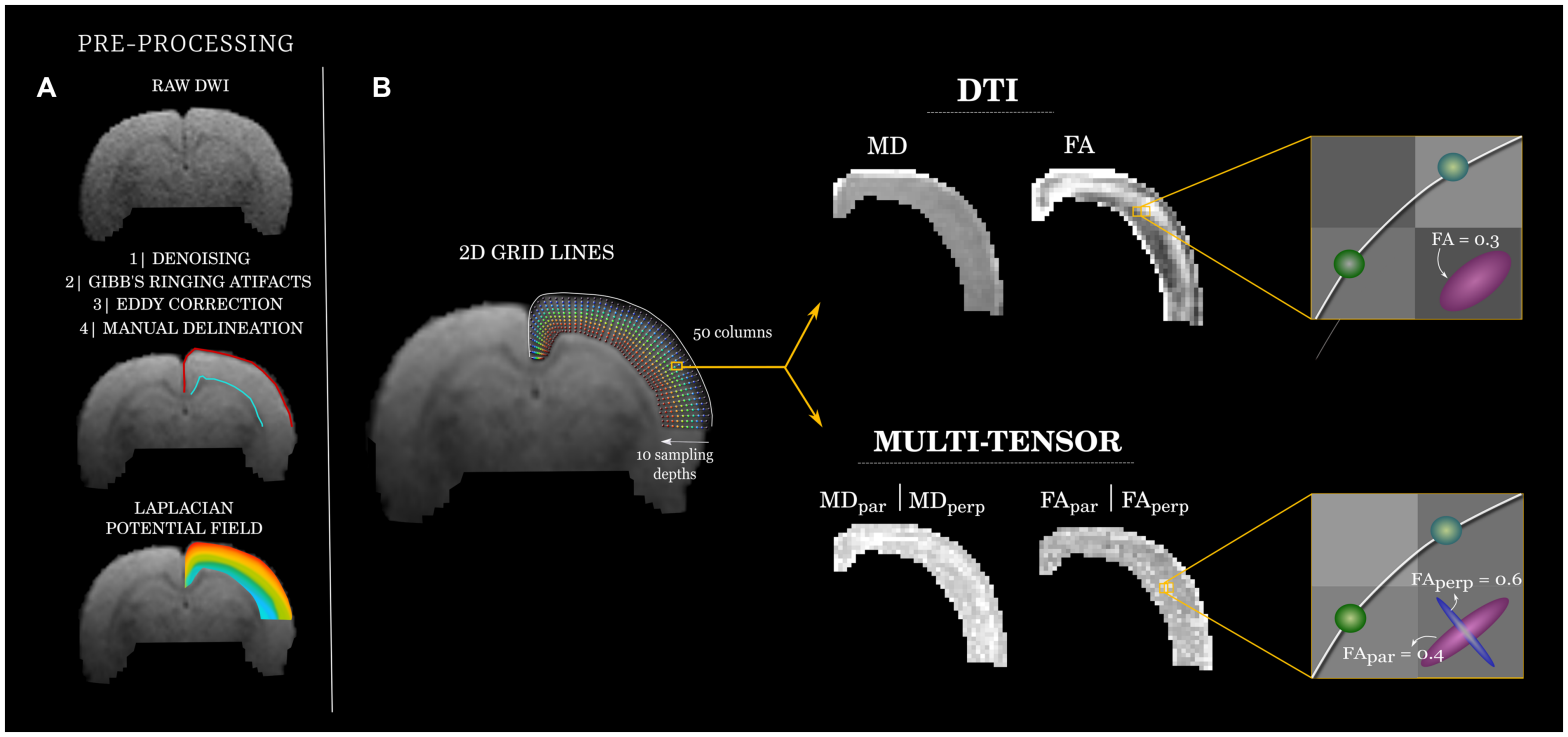
Diffusion analysis workflow. **(A)** Raw diffusion images (top, exemplary *b* = 650 s/mm^2^) were pre-processed and used to manually delineate (middle) the superficial (pial; red) and deep (gray/white matter transition; cyan) borders of the cortical mantle. A Laplacian potential field was estimated between the two cortical boundaries (bottom). **(B)** A coordinate system (left) was created for each rat by creating curved grid-lines that follow the Laplacian field (fifty equally-spaced origins at the pial boundary). Ten equally-distributed points across each grid-line are used to sample the quantitative dMRI maps, color-coded to indicate depth (blue: superficial; to red: deep). Exemplary DTI and MRDS (multi-tensor) maps are shown (middle). At each point of each grid-line, tensors fitted through MRDS were separated into parallel (_par_) and perpendicular (_perp_) to the grid-lines and their corresponding FA and MD values analyzed independently (right).

The dMRI data sets were first pre-processed. This included (1) denoising using random matrix theory (34), as implemented in MRtrix 3.0 (35) (dwidenoise). (2) removal of Gibbs-ringing artifacts using the method of local subvoxel-shifts proposed by (36), also implemented in MRtrix 3.0 (mrdegibbs) and (3) EPI susceptibility distortion correction through linear registration of all volumes to the main non-diffusion-weighted image using FLIRT version 6.0 from the FSL 6.2.0 library (37). We used the multi-resolution discrete-search method (MRDS) (28) which allows for the identification of one or more tensors within any given voxel (a Singularity container is available upon request). In the MRDS implementation, identified tensors were considered independent if their main eigenvectors formed an angle of at least 20 degrees. The maximum possible number of tensors per voxel was three, and the number of tensors that best explained the recorded signal was determined using the F-test per voxel. The conventional metrics can be then derived for each tensor, including fractional anisotropy (FA), and mean diffusivity (MD), which we separated according to their orientation (see below). We also included the diffusion tensor model DTI (26) as a baseline method due to its widespread use (Figure 1).

For each animal, the cortical slice at the level of the dorsal hippocampus was selected for further analyses. To have a common anatomical descriptor of the cortex, we fitted a curvilinear 2D grid to the cortical ribbon of the left hemisphere. This was achieved by manually delineating the brain pial surface and the junction of the gray and white matter using ITK-SNAP (38). Next, a Laplacian potential field was computed between these two boundaries using *minclaplace* (39). Laplacian curved lines were anchored at 50 points distributed along the pial boundary, which extended toward the gray/white matter junction (88 μm steps) describing slightly curved trajectories that follow the cortical anatomy. The resulting curved lines (henceforth referred to as grid-lines) were resampled to have 10 vertices each, thus providing a similar sampling scheme of the cortical depth irrespective of its thickness. Maps of DTI and MRDS metrics were sampled at each point of every grid-line using linear interpolation of the underlying data. Tensors derived from MRDS were labeled as being parallel or perpendicular to the grid-lines by computing the inner product of the (normalized) main eigenvector of each identified tensor and the (normalized) corresponding grid-line segment. The tensors yielding the highest and lowest dot products with respect to the grid-line segment were deemed as parallel and perpendicular to the grid-line, respectively (the orientation of tensors with respect to the imaging plane was not considered for the labeling). We refer to the corresponding metrics (i.e., FA, MD) with subscripts (_par_) for parallel and (_perp_) for perpendicular to the grid-lines, respectively. The routines described here are accessible at https://github.com/lconcha/Displasias.

### Statistical analysis

The spatial distribution of cyto- and myelo-architecture likely impacts the spatial distribution of diffusion metrics. Thus, at each point in the curvilinear grid and for each metric, we estimated between-group differences. Statistical significance was estimated with permutation tests at each point (5,000 permutations). Cluster-wise statistical inference was performed by computing the empirical null distribution of cluster sizes by tallying the size of resulting clusters after 5,000 grid-wise permutations (cluster-forming threshold: *p* < 0.05; four-point connectivity). Effect size was estimated with Cohen’s *d* at each point.

### Immunofluorescence

To investigate the histological features that drive changes of the diffusion profiles in the presence of cortical malformations, we performed immunofluorescent analyses of the myelo-architecture in a separate sample of 4 animals. At post-natal day 30, control and BCNU animals were intracardially perfused with 0.9% NaCI followed by 4% paraformaldehyde (PFA) solution. Brains were removed and post-fixed in fresh 4% PFA solution for 24 h. Then, specimens were immersed in sucrose solution 20% for 48 h followed by sucrose solution 30% for another 48 h, and stored at −80°*C. prior* to the histological procedure, coronal sections (20 μm-thick) of the cerebral region of interest were cut in a cryostat microtome (Leica) near to Bregma region (5.86–3.14 mm) and stored in cold phosphate buffer solution (PBS; Sigma-Aldrich) 1X. Blocking solution was performed using Bovine Serum Albumin (BSA; Sigma-Aldrich) solution 2% + 0.3% triton X-100 (ThermoFisher) in PBS for 45 min. For the double immunofluorescence staining, primary antibodies anti-Myelin Basic Protein (MBP; 1:200; Sigma-Aldrich) and anti- Neurofilament (NF200; 1:200; Sigma-Aldrich) were incubated for 24 h at 4°C. Then, slices were rinsed 3 times for 10 min in PBS 1X. Fluorescently tagged secondary antibodies (Molecular Probes, AlexaFluor, goat anti-mouse −488 and goat anti-rabbit −555; diluted at 1:500 in blocking solution) were incubated for 4 h at 4°C, rinsed 3 times for 10 min in PBS 1X and cover-slipped with microscope cover glass.

Full brain slices at the same level as the dMRI were captured using an Apotome-Zeiss fluorescence microscope with 488 and 565 nm emission filters, connected to a computer adapted with the AxioVision software (ver. 4.8) where the MosaiX module was used to capture mosaic images with a 10X objective. Structure tensor analysis (Puspoki 2016) was performed as implemented in OrientationJ (plugin available in https://github.com/Biomedical-Imaging-Group/OrientationJ). Pixel-wise structure tensors were computed from a local neighborhood with a specific Gaussian-shaped window of 15 μm, from which we derived local orientation, anisotropy and local coherency maps.

## Results

### Analysis of dMRI

**Figure 2:**
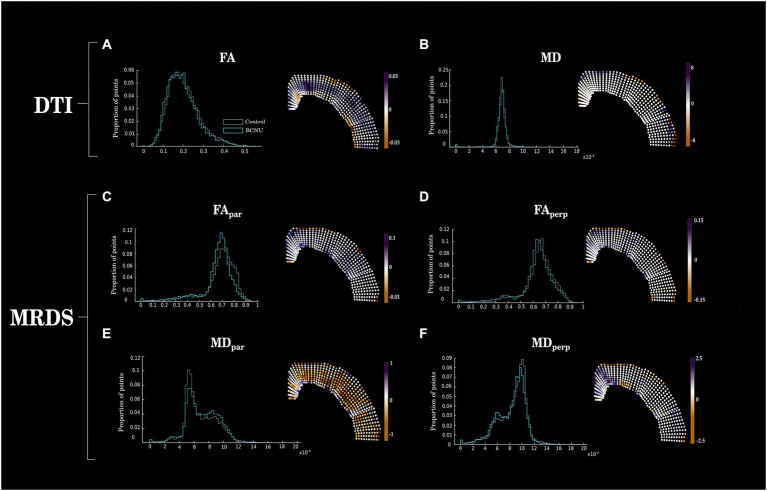
Between-group differences of DTI and MRDS. Histograms (left column) show mean values for each group, disregarding spatial information. Right column shows the spatially-organized mean difference between groups (Control–BCNU). While histograms show similar distributions of diffusion metrics between groups, spatial analysis highlights focal abnormalities of BCNU-treated animals. Corresponding statistical analyses are shown in Figure 3.

**Figure 3:**
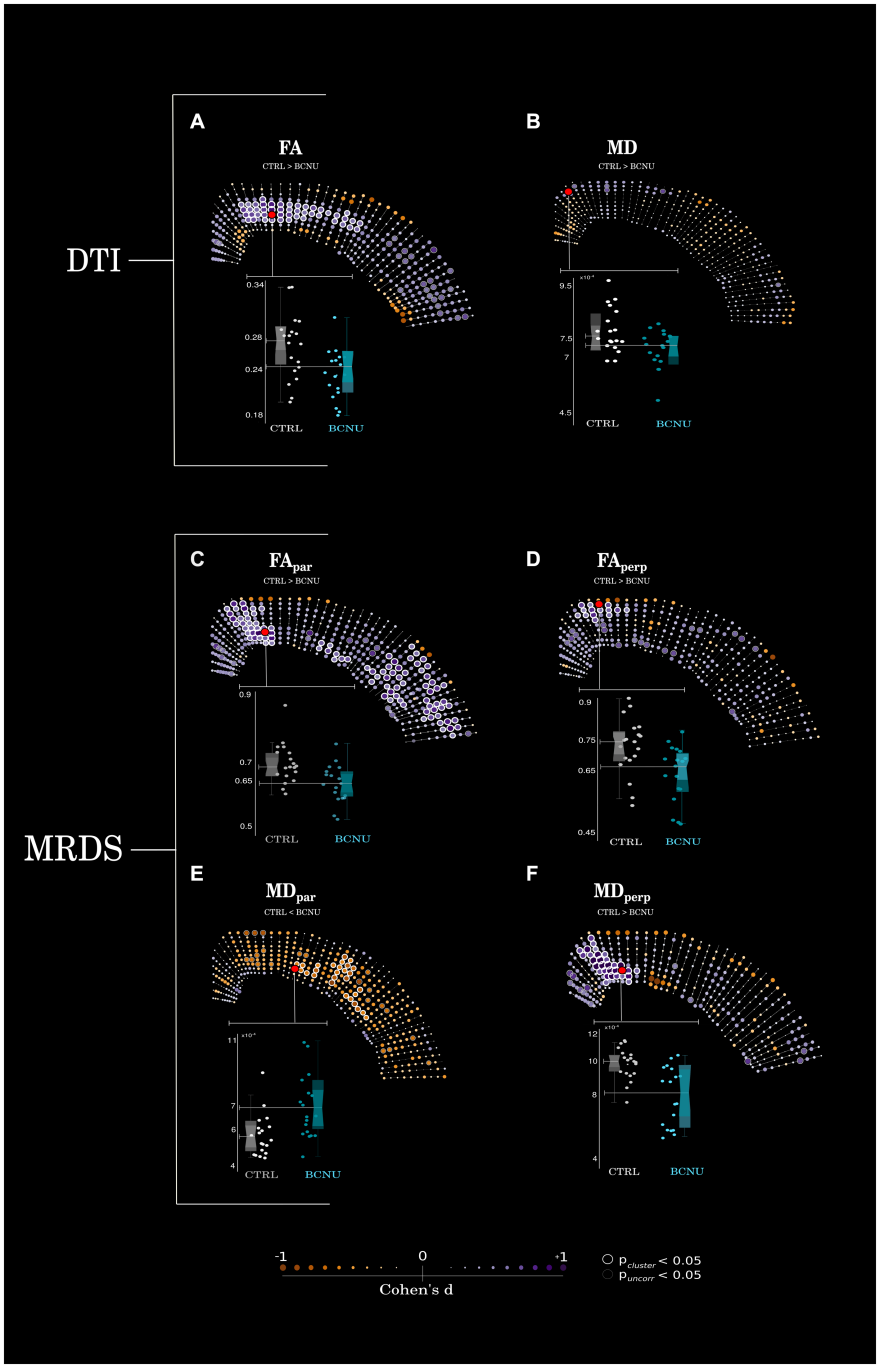
Statistical analyses. **(A)** Animals treated with BCNU showed reduced FA in the middle portions of retrosplenial and sensorimotor cortices, **(B)** no statistical differences were seen for MD. **(C–F)** Retrosplenial and motor cortices also showed reduced FA_perp_, FA_par_, and MD_perp_ with further reductions of FA_par_ and increased MD_par_ in the lateral cortex. Highlighted red dots denote the points of highest statistical significance, data from which are plotted as box plots (bottom). Between-group differences were assessed for statistical significance using permutation tests (point-wise uncorrected *p* < 0.05 shown as gray circles; cluster-wise inference shown as white circles for p_cluster_ < 0.05). Effect sizes (Cohen’s *d*) are shown as color and size of each point.

Histogram analysis of diffusion metrics were not sensitive to detect differences between control and experimental animals (Figure 2, left column). Spatial analyses using the curvilinear grid proved to be essential for the identification of focal diffusion changes (Figure 2, right column; Figure 3). Univariate statistics of diffusion metrics revealed between-group differences in several regions. The single diffusion tensor showed a large cluster of reduced FA of experimental animals in the middle third of the cortical depth across several regions including the retrosplenial cortex, primary motor cortex, parietal association cortex and primary somatosensory cortex (Figure 3A). There were also a few regions of reduced FA in the area corresponding to the secondary somatosensory cortex (p_uncorr_ < 0.05). Histogram and spatial maps for group-average AD and RD are shown in Supplementary Figure 1. MRDS identified three tensors in the majority of voxels across the cortical ribbon (Supplementary Figure 1). After separating said tensors into parallel and perpendicular to the grid-lines traversing the cortex, we identified a large cluster of reduced FA_par_ in the BCNU-treated animals, and the same metric was also reduced in large portions corresponding to the secondary somatosensory cortex, and the deepest regions of the primary somatosensory cortex, alongside increased MD_par_ (Figures 3C,E). Also, reduced MD_perp_ and FA_perp_ were identified in lesioned animals at the level of retrosplenial and primary motor, with FA_perp_ abnormalities spanning the entire depth of the cortex (Figures 3D,F).

### Histological evaluation

**Figure 4:**
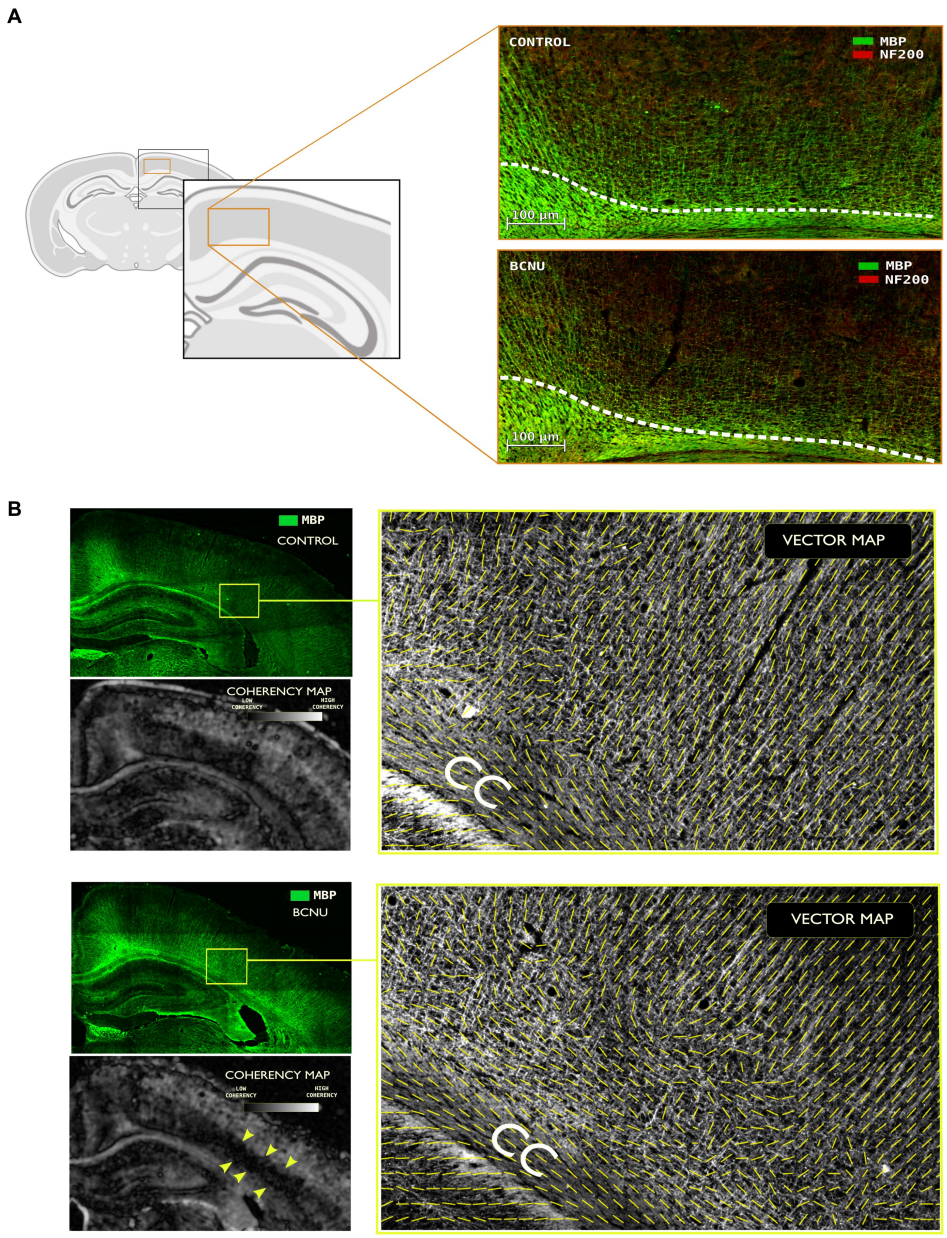
Immunofluorescence assessment with myelin basic protein (MBP) and neurofilament (NF200) primary antibodies. **(A)** Demyelination and disorganization of the myeloarchitecture are evident at the primary and secondary motor cortices. White line denotes the superior border of the cingulum. Note the decreased density of MBP-positive fibers in BCNU animals. **(B)** Structure tensor analysis of MBP was used to derive local texture coherency and vector maps. BCNU-treated rats showed low coherency at the level of layer V-VI (yellow arrows) and overall disorganized myeloarchitecture.

To study myeloarchitectural features, we performed immunofluorescence of MBP and NF200 of brain sections at the same level as dMRI evaluations of four different animals (2 rats per group). Figure 4 shows reduced myelination and disarrangement (MBP in green) of both tangential and radial fibers corresponding to M1 and M2. These findings co-locate with results derived from MD_perp_ and FA_par_ analysis. To further evaluate these histological features we used structure tensor analysis and computed a coherency map. This revealed loss of coherence (disorganization of myelin fibers) related to the sensorimotor cortex (S1) and particularly pointed at the level of V-VI layers. To evaluate fiber disorganization, we computed a vector map (15 μm Gaussian window) based on the texture of myelin fibers, which showed higher organization in Control animals when compared to BCNU.

## Discussion

Identification of subtle forms of focal cortical dysplasia remains a major challenge in a large group of patients with epilepsy, with negative impact on their surgical treatment. In this work, we provide evidence to support the ability of dMRI to assess the microstructural environment of the cortex, and its utility to detect microarchitectural abnormalities related to FCD in an animal model. Histological analysis revealed that diffusion abnormalities are related to altered coherence of myelinated intracortical radial and tangential fibers.

Most previous efforts to increase the visibility of FCD have focused on the imaging hallmarks of these lesions at the macroscopic level, such as increased cortical thickness, blurring of the gray/white matter interface, and increased relative intensity (12). Improvements on MRI acquisition allow for better visualization of FCD, and include the addition of sequences such as FLAIR or MP2RAGE (4), or the use of higher magnetic fields (40). Their automatic identification has been guided by statistical analyses of the aforementioned FCD imaging features either at the voxel or surface levels (41–44), with simultaneous analysis of different contrasts (e.g., T1 and T2 or FLAIR) providing better sensitivity (45). The advent of new methods based on machine learning and artificial neural networks have further pushed the ability of MRI to detect FCD (13, 16, 17), yet sensitivity remains around 80–90%, often at the expense of false positive findings (i.e., low specificity). These sophisticated analyses exploit what can be captured by anatomical imaging, but cannot look any further. As FCDs are inherently a histopathological pattern of cortical disorganization, and the macroscopic abnormalities may not capture the full extent of microscopic anomalies, imaging techniques that are able to resolve spatial organization at the sub-voxel level like the one used in this study, become relevant.

Diffusion-weighted MRI has proven useful for the characterization of tissue microstructure by querying the local microenvironment of water molecules within tissue (46). The white matter directly underlying FCD and other cortical malformations often displays diffusion alterations (47–49). Similar diffusion abnormalities can also be observed in deep white matter structures remote from the cortical lesions (50–53). Direct application of dMRI to study the cortex is less straightforward, as many assumptions for its interpretation do not hold true for the complex architecture of gray matter. Nonetheless, it is recognized that diffusion is not isotropic in the neocortex (19, 54, 55), that it varies as a function of age (54), that it is useful for the parcellation of brain regions (16, 20, 56–59), and that anisotropy is driven mostly by the mesoscopic organization of cellular components and unmyelinated neuropil (60). By disassembling the single diffusion tensor into components oriented radial and perpendicular to cortical microcolumns, McKavanagh et al. (61). showed associations with histological features that co-vary in presence of cortical degeneration due to multiple sclerosis, indicating potential use for dMRI as a tool to study cortical tissue in degenerative disorders. In recent years, multi-compartment biophysical diffusion models have been used for characterization of FCD. Reduced intracellular volume fraction, as inferred through neurite orientation dispersion and density imaging (NODDI), has been identified within dysplastic cortex in human patients (62, 63). Using tensor-valued dMRI, a novel acquisition scheme that simultaneously encodes diffusion in multiple directions, microscopic anisotropy demonstrated abnormalities in various malformations of cortical development, including FCD, that aid in the delineation of cortical lesions that exceed what is possible through conventional imaging such as T1 or FLAIR (64). Through the spherical mean technique (SMT) and systematic sampling of the cortical ribbon similar to our grid-line-based approach, Lorio et al. (62) demonstrated increased microscopic diffusivity throughout the depth of the cortex of patients with FCD, as compared to their homotopic regions. Although a known limitation of SMT is its misestimation of microscopic anisotropy (65), other diffusion metrics derived from NODDI were also abnormal in FCD tissue. However, both SMT and NODDI are unable to disentangle diffusion related to tissue components (also known as fixels) radial or tangential to the cortical surface, which we show to be differentially affected in FCD. While the single and multi-tensor models used here cannot separate diffusion compartments, our findings of reduced FA_par_ and FA_perp_, along with abnormal values of MD_par_ and MD_perp_ in the cortex of experimental animals (Figure 3) are in line with previous literature.

Animal models of FCD provide opportunities to test the sensitivity of different dMRI methods to cortical alterations, with different experimental approaches providing control over severity, extension, and temporal evolution of the cortical lesions. While genetic models of FCD targeted to the mTOR-signaling pathway are useful to understand the mechanisms of epileptogenesis of these cortical malformations (66), physical and chemical interventions provide good approximations to the histopathology seen in humans (67, 68). The BCNU model produces cortical alterations similar to those seen in FCD type IIa in humans, with disarrangement of the cortex, as well as dysmorphic and heterotopic neurons (69). Anatomical MRI of BCNU-treated rats has previously demonstrated macrostructural abnormalities, such as enlargement of the ventricles, cortical thinning, hippocampal hypoplasia, and agenesis of the corpus callosum (70). While our BCNU-treated animals displayed histopathological features of FCD (Figure 4), the previously-described gross anatomical malformations were not as marked, likely due to the relatively young age at which they were scanned. Contrast between cortical layers can be enhanced through systemic administration of Mn^2+^, with BCNU-treated animals displaying an abnormally flat intensity profile across the cortical depth, indicative of altered cortical organization (70). Toxic effects limit the application of Mn^2+^ as an exogenous contrast in humans, thereby hampering its use for the diagnosis of FCD, and fostering the use of endogenous sources of contrast for the study of cortical layering. In a rodent model of FCD that shares similarities with the one used here (49) reductions of FA were found in white matter structures, as well as in the retrosplenial and cingulate cortices that are consistent with our findings (Figure 3). In their study, alterations of cortical layering and hypomyelination were also identified. The abnormal myelin structure seen in our experimental animals (Figure 4) is in agreement with the reduced myelination patterns seen in human FCD specimens (71). However, rather than a drastic reduction of myelin, which likely could be detected using myelin-sensitive MRI methods (72), there is a disorganization of the fiber network, for which dMRI is particularly tailored.

The cortical structure is complex, but stereotypical. Considering intracortical fibers that run either tangential or perpendicular to the pial surface, we tested whether a multi-tensor approach could capture the spatial organization from these two fiber populations. MRDS was indeed able to identify sets of voxels spatially-grouped in regions with reduced FA or MD either parallel or perpendicular to the pial surface. Its simple biological interpretation and the lenient requirements for data acquisition (i.e., at least two *b* > 0 s/mm^2^ shells with modest radial sampling of q-space) suggest that this dMRI method could be suitable for identification of FCD in humans. Other approaches to evaluate dMRI may also prove beneficial, as recent reports have shown (62, 64). The explicit inclusion of the soma diffusion compartment in a biophysical model of the cortex extends the NODDI model and provides new insights into the cortex that match spatial patterns of cellular and fiber density (73), albeit with very high data acquisition requirements. With the recognition that time-dependence and exchange play important roles in the behavior of diffusion in the cortex (hitherto often ignored for analysis of white matter) (74), methods such as filter-exchange imaging (FEXI) (75) and neurite exchange imaging (NEXI) (76) are promising tools for the study of FCD.

There are limitations to the current study. First, the direct application of a multi-tensor model originally designed for the analysis of white matter to describe the cortex likely introduces bias in the estimation of the diffusion profile. MRDS has proven to robustly identify up to three tensors in areas of crossing fibers in white matter (28), but its reliability in gray matter has not been assessed. As the multi-tensor fitting method is a dMRI signal representation, rather than a biophysical model (32), our results show that it has the sensitivity to successfully detect changes in the cortical tissue, albeit without specificity to tissue components. Second, the two-dimensional image acquisition provided high in-plane resolution, but the thick slices (1 mm) surely introduce partial volume effects in all dMRI estimations. Similarly, while tensors parallel to the grid-lines were reliably identified, tensors perpendicular to the grid-lines included those with either in-plane orientation, as well as tensors oriented perpendicular to the imaging plane (i.e., related to fibers running rostro-caudally). Third, the study lacks direct correlation between dMRI metrics and corresponding histological features from the same specimens, which would have provided a direct interpretation of the diffusion alterations seen with MRDS and DTI. The three-dimensional architecture of the cortical ribbon is not readily apparent on two-dimensional histological sections (Figure 4), and the relation between rostro-caudal tangential fibers and tensors identified as perpendicular to the grid-lines is obscured. Moreover, tissue components not explored here (e.g., glial cells) may also play an important role in the modulation of diffusion in the presence of FCD. Fourth, spatial resolution will be a hurdle for the applicability of our method for *in vivo* human dMRI. Spatial resolution of dMRI in humans is typically 2x2x2 mm^3^ (although 1x1x1 mm^3^ and below is possible) on conventional clinical scanners. This means that the cortical ribbon contains only 2–4 voxels that can be sampled. Nonetheless, as other groups have shown (62, 77), dMRI metrics can be sampled as a function of cortical depth, albeit with less spatial resolution than the one we report in our animal data. Further, multi-tensor fitting (and other per-bundle diffusion analysis methods) is able to disentangle the two main fiber populations within the cortex (i.e., aligned with cortical columns or tangential to the cortical surface), which holds valuable information regarding tissue organization even at relatively low spatial resolution. Despite these limitations, MRDS proved useful in this study for the identification of abnormalities and their separation of those related to ascending/descending fibers and those related to tangential intra-cortical fibers without requiring extremely high *b* values or long acquisition times.

Overall, our findings provide justification for the use of dMRI (and MRDS) for the identification of alterations of cortical architecture and could be used in clinical applications for the identification of FCD in patients undergoing evaluation for medically-refractory focal epilepsy.

## Data availability statement

The raw data supporting the conclusions of this article will be made available by the authors, without undue reservation.

## Ethics statement

The animal study was reviewed and approved by Ethics Committee, Institute of Neurobiology, Universidad Nacional Autónoma de México.

## Author contributions

PV, DC-S, HL-M, AR-M, and LC contributed to the conception and design of the study. PV, DC-S, AP-M, AR-M, and LC performed the analyses. HL-M, DC-S, and AA performed the experiments and imaging. PV performed the histological procedures and structure tensor analysis. JL-S supervised the histological analyses. RC-L provided code and supervised analysis of diffusion. PV and LC wrote the first draft of the manuscript. All authors contributed to the manuscript revision, read, and approved the submitted version.

## Funding

This work was provided by UNAM-DGAPA- PAPIIT (IN204720 and IA200621) to LC.

## Conflict of interest

The authors declare that the research was conducted in the absence of any commercial or financial relationships that could be construed as a potential conflict of interest.

## Publisher’s note

All claims expressed in this article are solely those of the authors and do not necessarily represent those of their affiliated organizations, or those of the publisher, the editors and the reviewers. Any product that may be evaluated in this article, or claim that may be made by its manufacturer, is not guaranteed or endorsed by the publisher.

## Supplementary material

SUPPLEMENTARY FIGURE Group average values for axial and radial diffusivities (AD, RD, respectively), and number of tensors identified by MRDS (first two columns). The same maps are shown as histograms on the right (third column). The fourth column illustrates the absolute mean difference between the two groups, and the corresponding statistical analyses and effect sizes are shown on the right-most column.
