# Supplementary figures and images for: Multi-tensor diffusion abnormalities of gray matter in an animal model of cortical dysplasia
Source: Front Neurol. 2023 May 5;14:1124282. doi: 10.3389/fneur.2023.1124282 (PMC10278582; doi:10.3389/fneur.2023.1124282)

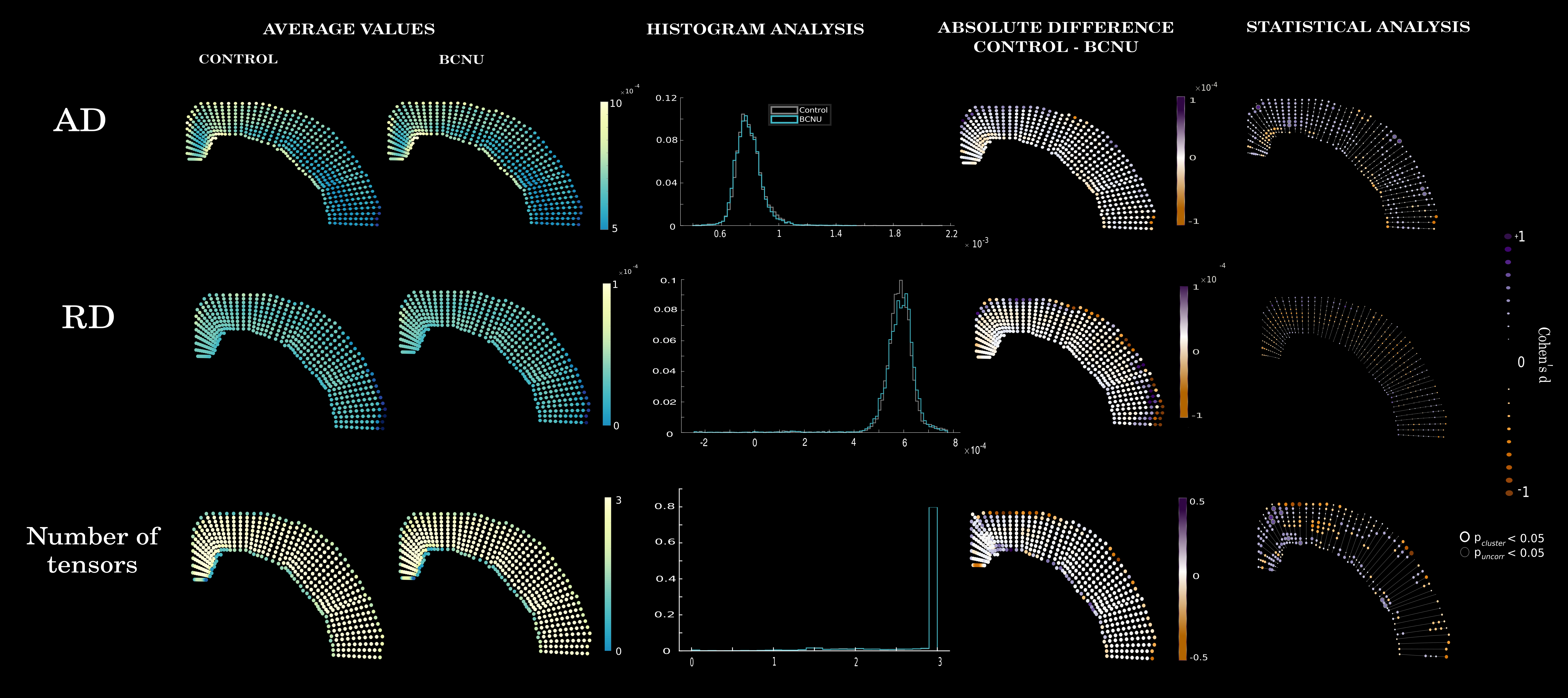

Supplement: SUPPLEMENTARY FIGURE — Group average values for axial and radial diffusivities (AD, RD, respectively), and number of tensors identified by MRDS (first two columns). The same maps are shown as histograms on the right (third column). The fourth column illustrates the absolute mean difference between the two groups, and the corresponding statistical analyses and effect sizes are shown on the right-most column. [file Image_1.TIF]
